# A Novel Optical Quantal Analysis of Miniature Events Reveals Enhanced Frequency Following Amyloid β Exposure

**DOI:** 10.3389/fncel.2020.564081

**Published:** 2020-11-03

**Authors:** Henry B. C. Taylor, Rudi Tong, Alexander F. Jeans, Nigel J. Emptage

**Affiliations:** ^1^Department of Pharmacology, University of Oxford, Mansfield Road, Oxford, United Kingdom; ^2^Montreal Neurological Institute and Hospital, Montreal, QC, Canada

**Keywords:** pHluorin, neurotransmitter release, mEPSC, Alzheimer’s, presynaptic, miniature neurotransmission

## Abstract

Non-evoked miniature release of neurotransmitters is increasingly recognized as playing an important role in neural function and is implicated in synaptic plasticity, metaplasticity, and homeostasis. Spontaneous miniature release events (minis) are usually measured electrophysiologically by recording the miniature postsynaptic currents (mEPSCs) that they evoke. However, this indirect technique can be confounded by changes within the postsynaptic neuron. Here, using the fluorescent probe SynaptopHluorin 2×, we have developed an optical method for the measurement of minis that enables direct assessment of release events. We use the technique to reveal that the frequency of minis following incubation of hippocampal neurons with Amyloid β oligomers (Aβo) is increased. Electrophysiological mEPSC recordings obtained under the same conditions report a decrease in frequency, with the discrepancy likely due to Aβo-induced changes in quantal size. Optical quantal analysis of minis may therefore have a role in the study of minis in both normal physiology and disease, as it can circumvent potential confounds caused by postsynaptic changes.

## Introduction

It is well documented that, in addition to neurotransmitter release evoked by action potential (AP) invasion into boutons, neurons occasionally release vesicles of neurotransmitter in a non-evoked or spontaneous manner. Until quite recently presynaptic “miniature release events” (minis) were thought to be functionally unimportant, however, it is now clear that they serve distinct physiological roles. Miniature release mechanisms are, in part, separate from those of evoked release (Deitcher et al., 1998), and minis appear to have partially distinct receptor targets (Atasoy et al., 2008; Reese and Kavalali, 2016) and release machinery (Ramirez et al., 2012) to evoked release. Spontaneous miniature excitatory postsynaptic currents (mEPSC) amplitude and frequency reflect previous activity at the synapse (Turrigiano et al., 1998; Liu et al., 2000; Bacci et al., 2001), and may play a role in metaplasticity, with increased mEPSC amplitude and frequency accompanying long-term potentiation (LTP; Oliet et al., 1996) and lowered mEPSC amplitude and frequency correlating with the strength of long-term depression (LTD) induction (Zhang et al., 2005). Also, minis have been implicated in homeostatic synaptic scaling (Pozo and Goda, 2010; Turrigiano, 2012; Kavalali, 2015; Gonzalez-Islas et al., 2018) *via* the regulation of dendritic protein synthesis (Sutton et al., 2004, 2006; Sutton and Schuman, 2006). Synaptic scaling is impaired in certain neurodegenerative diseases such as Alzheimer’s disease (AD; Frere and Slutsky, 2018), and indeed minis appear to be altered in AD. Following exposure to disease-relevant assembly states and concentrations of the AD-associated peptide amyloid β (Aβ), in particular highly pathogenic Aβ oligomers (Aβo; Viola and Klein, 2015), multiple studies have shown that mini frequency is reduced (Kamenetz et al., 2003; Shankar et al., 2007; Nimmrich et al., 2008; Talantova et al., 2013), although in one study this reduction was preceded by a short-lived frequency increase (Parodi et al., 2010). These Aβo-induced changes in mini frequency have usually been explained by a presumed presynaptic weakening over time, although there is no direct evidence for this. There is, however, a well-established process of postsynaptic weakening and depression which begins rapidly following the addition of Aβo (Sheng et al., 2012).

Minis are typically studied experimentally by recording the currents that they produce (mEPSCs) at the soma of the postsynaptic neuron. This approach introduces some important confounds into the measurements. First, it is not possible to attribute events measured at the soma to specific synapses, precluding the study of a specific contribution of minis at a specific site, or those arising at clusters of synapses on specific axonal or dendritic branches. The latter may be particularly important as there is clear evidence that dendritic branches are an important spatial unit for synaptic regulation (Branco et al., 2008) and information storage (Govindarajan et al., 2011). Second, electrical signals from distal synapses can be attenuated by the time they reach the soma due to the passive cable properties of dendrites. This will produce a sampling bias towards synapses on proximal dendritic branches in electrophysiological recordings (Williams and Stuart, 2003). Finally, changes in postsynaptic strength, particularly weakening, can occur as a result of physiological processes such as LTD, or as a consequence of pathology, as in Aβo addition. These changes will affect the amplitude of mEPSCs and, if postsynaptic weakening is significant, could cause some events to fall below the detection threshold of the recording. Under these circumstances lost events reduce the total mini count and will generate mini frequency measurements that are artefactually low.

Direct imaging of presynaptic terminals offers the opportunity to circumvent these problems. There have been successful attempts to optically measure quantal release events, both evoked and spontaneous, using the fluorescent dye FM 1–43 (Ryan et al., 1997; Tokuoka and Goda, 2008), but very few attempts have been made to look specifically at miniature release. Here, we demonstrate a novel, optical method of directly measuring miniature presynaptic release events using the genetically encoded fluorescent probe SynaptopHluorin 2× (SypH 2×). We show that this circumvents the confounds associated with postsynaptic weakening and allows accurate changes in the frequency of miniature release to be measured following Aβo exposure. Given the importance of minis in the regulation of synaptic strength this technique should facilitate study in several areas of synaptic biology.

## Materials and Methods

### Primary Neuronal Culture and Transfection

Hippocampal neurons from postnatal day 1 (P1) Wistar rats were seeded onto poly-D-lysine-coated coverslips and cultured in Neurobasal medium A supplemented with 2% fetal bovine serum (FBS), 2% B27 Plus, 1% Glutamax, and 1% penicillin/streptomycin. The day after plating, half the medium was exchanged for Neurobasal medium A supplemented with 2% B27 Plus and 1% Glutamax only, which was used for all subsequent feeds. Cells were transfected at 8 days *in vitro* (DIV) with SypH 2× (gift of Dr. Y. Zhu) plasmids using Lipofectamine 2000 (Invitrogen). To investigate the effects of Aβo, cells were incubated in Aβo [200 nM; prepared according to a well-validated protocol (Klein, 2002)] for over an hour in the incubator.

### Aβo Synthesis

Experiments were conducted using a single batch of Aβ_1–42_ peptide (Abcam) and oligomers were synthesized according to a validated protocol (Klein, 2002). This widely-used and highly reproducible preparation has previously been characterized in detail and found to contain predominantly the low molecular weight species most associated with synaptotoxicity (Lambert et al., 2007; Velasco et al., 2012). Briefly, solid Aβ_1–42_ was dissolved in cold hexafluoro-2-propanol (HFIP; Sigma Aldrich). The peptide was incubated at room temperature for at least 1 h to establish monomerization and randomization of the structure. The HFIP was aliquoted and allowed to evaporate overnight, followed by 10 min in a Savant Speed Vac. The resulting peptide was stored as a film at −80°C. The film was dissolved in anhydrous dimethylsulfoxide (DMSO; Sigma Aldrich) to 5 mM, diluted to approximately 100 μM with Ham’s F12 (without phenol red, with glutamine; Caisson Laboratories, Logan, UT) and briefly vortexed. The solution was incubated at 4°C for 22–24 h, and soluble oligomers obtained by centrifugation at 14,000 *g* for 10 min at 4°C.

### Live Cell Imaging

Experiments were performed on dissociated hippocampal cultures prepared as described above at DIV 14–18 when synapses have matured. Coverslips were mounted in a Chamlide EC-B18 stimulation chamber on the stage of an Olympus IX-71 inverted microscope fitted with a 100×, NA 1.40 UPlanSApo objective and an Andor iXon EM CCD camera. Fluorescence illumination was supplied by a 100 W mercury lamp used with appropriate neutral density filters and shuttered (Uniblitz CS25, Vincent Associates) during all non-data acquisition periods. Suitable fields of boutons were selected using the readily visualized resting fluorescence signal exhibited by SypH 2×, the additional criterion being that boutons show normal, healthy morphology with a regular outline, and relatively modest resting fluorescence signal that increases following NH_4_Cl unquenching. When required, APs were evoked by passing 20 V, 1 ms current pulses from a custom-made stimulation box *via* platinum electrodes. We have previously shown that each of these 20 V pulses has a greater than 97% probability of eliciting a single AP in stimulated neurons in our experimental setup (Jeans et al., 2017). Imaging of pHluorins was carried out at 1 Hz, with stimulation, image acquisition, and shuttering all under the coordinated control of WinWCP software (Strathclyde Electrophysiology Software). Experiments were carried out at room temperature in Tyrode’s solution (120 mM NaCl, 2.5 mM KCl, 20 mM HEPES, 30 mM glucose, 2 mM CaCl_2_, 2 mM MgCl_2_, pH 7.4) with APV (50 μM) and NBQX (10 M) to block recurrent activity. TTX (1 μM) was added for experiments investigating minis. NH_4_Cl unquenching was achieved by replacing Tyrodes solution with a Tyrodes solution containing 50 mM NH_4_Cl in substitution of 50 mM NaCl. For experiments examining minis, folimycin (10 nM) was added after 10 baseline images (Figure 2A). For experiments using nicotine, this was present (20 μM) in the Tyrode’s solution. Where cells have been incubated in Aβo, this is also present during the experiment.

**Figure 1:**
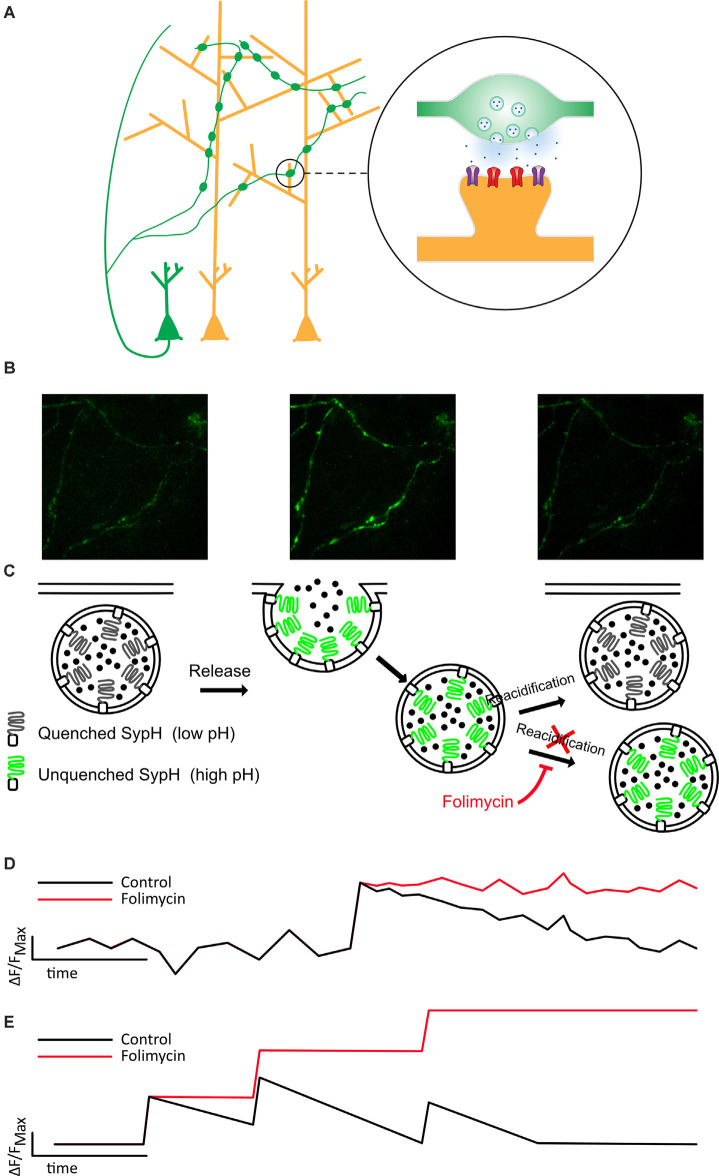
Folimycin allows the summation of SypH 2× signals from multiple miniature release events. **(A)** Schematic illustrating the presynaptic expression of SypH 2× in boutons of dissociated neurons (green) that form synapses on postsynaptic dendrites of other neurons (yellow). **(B,C)** Top images show representative fields from a SypH 2×-expressing neuronal culture during a typical experiment. The left panel shows SypH 2× fluorescence quenched at baseline before vesicle exocytosis occurs (middle panel) with accompanying unquenching of SypH 2× fluorescence as it is exposed to the neutral pH of the extracellular milieu. Following this, vesicles are reclaimed by endocytosis and as these are reacidified, fluorescence is once again quenched. These phases are represented in the schematic images below, which also demonstrate how the cell-impermeable inhibitor of vesicular reacidification folimycin can be used to block the quenching of endocytosed vesicles so that the final signal change at a bouton reflects all vesicles released during the experiment. **(D)** This schematic cartoon of a fluorescence signal at a bouton following a single release event with (red) and without (black) folimycin demonstrates how preventing vesicle reacidification prevents the SypH 2× fluorescence signal from being quenched, ensuring that the total signal change will reflect a sum of all vesicles released during the experiment, as shown in **(E)**.

**Figure 2:**
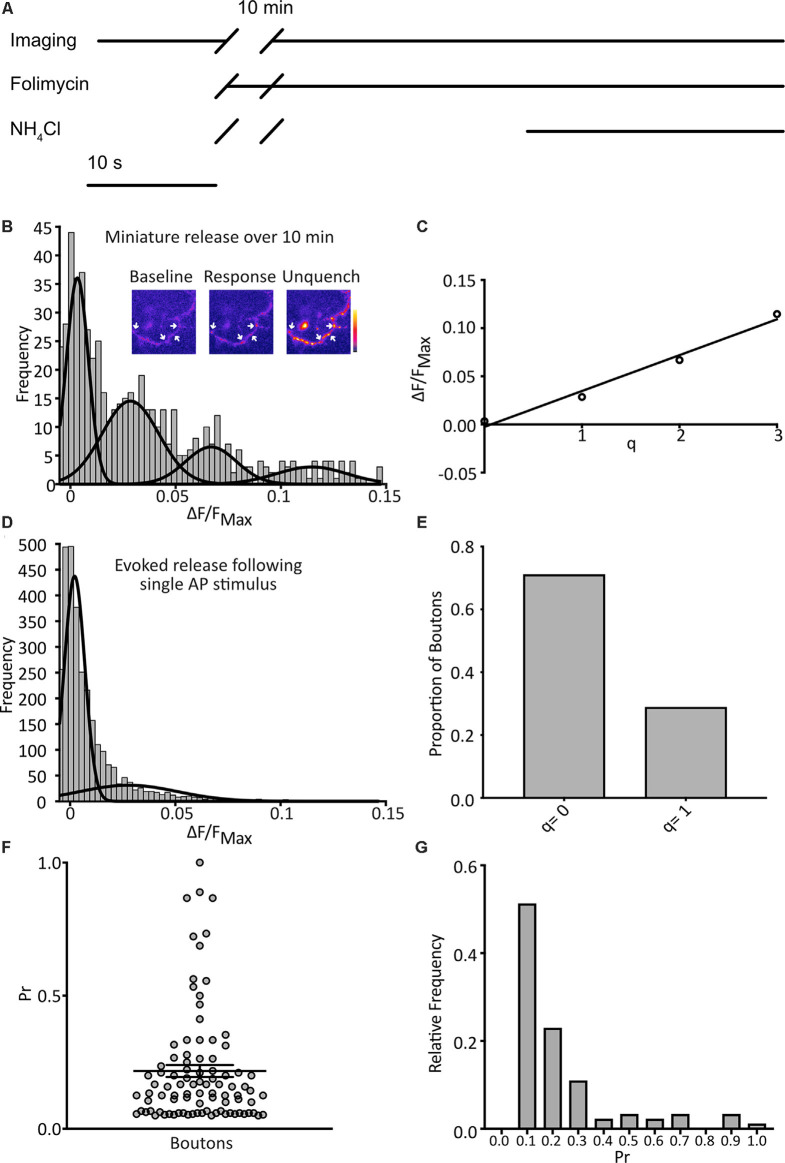
Detection of quantal spontaneous miniature release. **(A)** Experimental scheme with baseline images taken before the addition of the vesicular ATPase inhibitor, folimycin, and 10 min wait, before further image acquisition and unquenching using basic NH_4_Cl-containing buffer. TTX is present throughout. **(B)** Following a 10 min incubation in TTX and folimycin, a frequency distribution of responses, calculated for each bouton as the background-subtracted total signal change over the 10-min incubation normalized to the maximum signal change following NH_4_Cl addition, was plotted (*n* = 574 synapses from 31 coverslips). Multiple Gaussian curves were fit as described in the main text. Inset: a representative field showing boutons (arrows) at different points of an experiment. **(C)** The mean of each Gaussian was plotted against the number of release events it represents and after linear regression fitting the interpeak distance, a measure of quantal size (q), was estimated as the slope (*q* = 0.037 ΔF/F_NH_4_Cl_). **(D)** Responses of boutons to 1 action potential (AP) stimuli with a frequency distribution of responses (*n* = 4297 observations from three coverslips) and an objective Gaussian curve fitting algorithm was used to model the distribution with a mean of the second Gaussian giving *q* = 0.028. **(E)** Proportion of observations contributing to each Gaussian (*q* = 0: 0.79; *q* = 1: 0.21). **(F)** Pr of individual boutons over the experiment (*n* = 92 boutons from three coverslips, mean Pr = 0.22 ± 0.02). **(G)** frequency distribution of Pr derived from data presented in **(E)**.

### Patch Clamp Recording of mEPSCs

mEPSCs were recorded from dissociated hippocampal cultures at DIV 14–18. These recordings were carried out in whole-cell voltage-clamp, holding cells to −70 mV, using patch electrodes (5–8 MΩ) filled with Cs internal solution (135 mM CsCl, 2 mM MgCl_2_, 2 mM Na_2_-ATP, 0.2 mM Na-GTP, 10 mM HEPES). The internal solution was allowed to diffuse for 2 min before mEPSCs were recorded using an Axopatch 2A amplifier (Axon Instruments) and WinWCP software (Strathclyde Electrophysiology Software). Recordings were then analyzed using Mini Analysis from Synaptosoft, and mEPSCs were detected manually.

### Analysis of Imaging

Boutons were selected for analysis using a 2 μm diameter ROI. Experiments imaging pHluorins were analyzed using a custom-written MATLAB script. For analysis of minis, background fluorescence was subtracted from data points, which were then normalized to the bouton’s NH_4_Cl signal to allow for changes in expression of SypH 2× between boutons. The difference in fluorescence between baseline and 10 min after folimycin incubation was then calculated. Responses of each bouton were classified into bins of fixed-width, and this binned data were used to plot a frequency distribution. A Gaussian mixture model was then fitted to the (unbinned) data, with the optimal number of Gaussians determined by the model resulting in the lowest Akaike Information Criterion (AIC). The Gaussian distributions were ranked according to their expected values, with the rank corresponding to the number of release events (q). Having identified the boundaries (defined as the intersections between Gaussian distributions), we assigned each bouton within those boundaries as having undergone the number of miniature release events to which that distribution corresponds. To compare different conditions with different numbers of boutons, the relative proportions of boutons in each Gaussian distribution rather than the absolute numbers were used.

For one AP experiment, data points were background adjusted as before, and for each simulation, a response was measured as a response (average two points after stim) - baseline (average of first five points). These were then normalized to the NH_4_Cl signal. A frequency distribution was then generated in the same range as before, and two Gaussians fitted. Having found the intersection of the Gaussians, observations below the intersection were designated as not released, while those above were assigned as a release event. Probability of release (Pr) was then calculated for each bouton, discarding any bouton that did not release at all or had fewer than 15 usable measurements.

For 100 AP experiments, data points were background adjusted and normalized to the NH_4_Cl signal as before. The maximum response was calculated as the average of two points immediately following the end of stimulation minus the average of the first eight baseline time points.

## Results

### SypH 2× Allows Direct Measurement of Miniature Release in the Presence of Folimycin

To directly probe release, we used SypH 2×, a genetically encoded, presynaptically expressed reporter of vesicle fusion (Zhu et al., 2009) that fluoresces at neutral pH, and is quenched at the low pH observed within acidified synaptic vesicles (Figures 1A–C). SypH 2× and variants have been used to observe responses to single AP stimulation (Zhang et al., 2009; Jeans et al., 2017) given their enhanced signal-to-noise ratio due to the inclusion of multiple pHluorin moieties (Zhu et al., 2009). Despite this, it is difficult to resolve single release events from the background signal, particularly when seeking to detect events that are not time-locked to a stimulus. Therefore, we used the cell-impermeable vesicular ATPase inhibitor folimycin (Figure 1), which blocks reacidification of synaptic vesicles (Ertunc et al., 2007), thereby allowing events to be summed to produce a signal that is readily quantified (Figures 1C,D).

We imaged cultured hippocampal neurons expressing SypH 2× in the presence of tetrodotoxin (TTX; 1 μM) at pH 7.4 to isolate spontaneous miniature release events. Images were taken at 1 Hz over 10 s to give a baseline signal before the addition of folimycin (10 nM). After waiting for 10 min a series of images was again acquired at 1 Hz for 30 s. Over the last 10 s, SypH 2× was unquenched by alkalization using a buffer containing NH_4_Cl (pH 7.4) to reveal the maximal SypH 2× response at each terminal (Figure 2A). All responses were normalized to this maximal response to control for inter-terminal differences in pHluorin expression level (Jeans et al., 2017). Background adjusted responses were then plotted as a frequency distribution. Using a control dataset, the AIC was determined for Gaussian mixture models with varying numbers of Gaussian distributions (1–10 Gaussians) fitted to the data. The lowest (optimal) AIC value corresponded to a fit of four Gaussians (Figure 2B). The expected values of these distributions were equally spaced, as would be anticipated if events were quantal. Indeed, the resultant plot fitted well with a linear relationship (*R^2^* = 0.98), with the interpeak distance representing quantal size (q; *q* = 0.037 ΔF/F_NH_4_Cl_; Figure 2C).

To validate our calculated value for q we studied evoked release events following single AP stimuli. We serially imaged SypH 2×-expressing boutons over 10 s while giving a single stimulus, subjecting each to 23 trials with 30 s rest between each one. Using the same equipment and conditions, we have previously confirmed that >97% of delivered stimuli elicit a single AP in stimulated neurons (Jeans et al., 2017). We plotted a frequency distribution of the NH_4_Cl-normalized responses and fitted this with two Gaussian distributions (corresponding to either no release or one release event) using a custom-written objective curve fitting script. We again derived q as the interpeak distance (Figure 2D). This yielded a *q* estimate of comparable magnitude to that derived from the mini-experiment (*q* = 0.028 ΔF/F_NH_4_Cl_). The proportion of events that showed successful release was 21%, corresponding to an average probability of release across all events of 0.21 (Figure 2E). Next, we examined the properties of the individual boutons, calculating the probability of release (Pr) as the proportion of events where the release occurred for that bouton over the 23 trials, and discarding silent boutons. We plotted these values as a frequency distribution (Figure 2G) with a mean Pr of 0.22 and a median of 0.14, which is highly consistent with that observed by others in cultured hippocampal neurons (Branco et al., 2008; Tokuoka and Goda, 2008). Overall, these results indicate that our method can detect quantal miniature release events with high sensitivity.

### SypH 2× Detects Miniature Release Frequency Changes, and Aβo Incubation Increases the Frequency of Miniature Release Events

**Figure 3:**
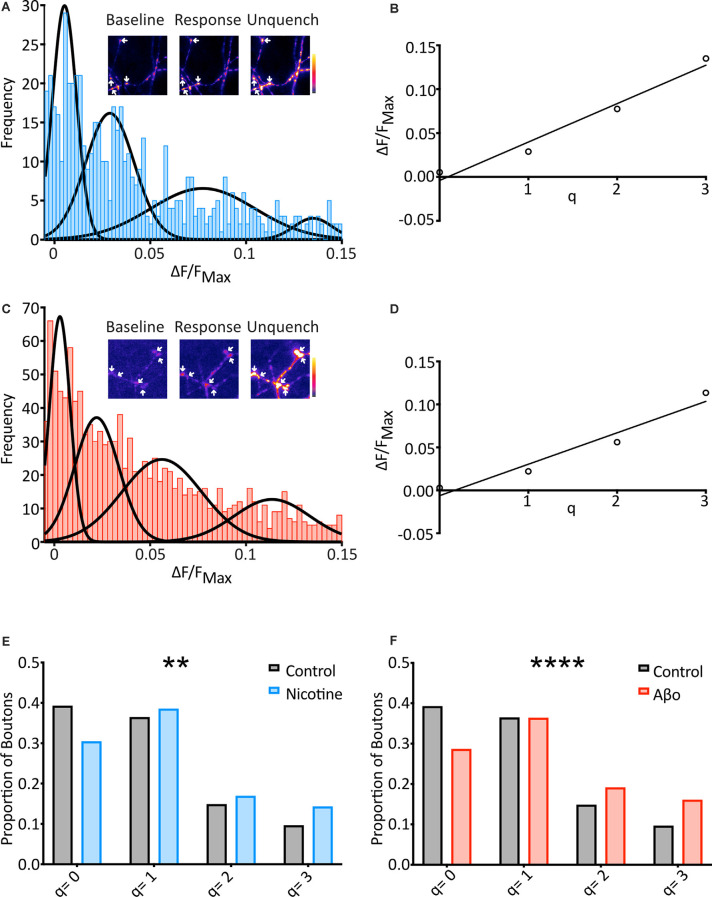
SypH 2× can detect changes in mini frequency, and Aβo treatment increases the frequency of optically-detected miniature events. **(A)** Cells were subjected to the same protocol as previously (see Figure 2A) in the presence of nicotine (20 μM) and frequency distribution of responses were plotted (*n* = 569 synapses from 21 coverslips). Multiple Gaussians were fit as for control. Inset: a representative field showing boutons (arrows) at different points of an experiment. **(B)** Linear regression fitting of a plot of the mean of each Gaussian vs. number of release events allowed an estimation of quantal size (q) as the slope of the plot (*q* = 0.044 ΔF/F_NH_4_Cl_). **(C)** Aβo-incubated cells were also subjected to the protocol shown in Figure 2A, and the frequency distribution of responses was plotted (*n* = 1217 synapses from 57 coverslips). Multiple Gaussians were fit as for control. Inset: a representative field showing boutons (arrows) at different points of an experiment. **(D)** Linear regression fitting of a plot of the mean of each Gaussian vs. number of release events allowed an estimation of quantal size (q) as the slope of the plot (*q* = 0.037 ΔF/F_NH_4_Cl_). **(E)** The proportion of boutons contributing to each Gaussian for control (black/gray) vs. nicotine (blue: Control: 39.2% of boutons not releasing, 36.4% of boutons releasing 1 quantum, 14.8% of boutons releasing 2 quanta, 9.6% of boutons releasing 3 quanta, *n* = 574 synapses from 31 coverslips; Nicotine: 30.4% of boutons not releasing, 38.5% of boutons releasing 1 quantum, 16.9% of boutons releasing 2 quanta, 14.2% of boutons releasing 3 quanta, *n* = 569 synapses from 21 coverslips). Note the control data-set is that shown in Figure 2. **(F)** Proportion of boutons contributing to each Gaussian for control (black/grey) vs. Aβo-incubated (red). The control data-set is that shown in Figure 2 and used in **(E)** above (Aβo: 28.6% of boutons not releasing, 36.3% of boutons releasing 1 quantum, 19.1% of boutons releasing 2 quanta, 16.0% of boutons releasing 3 quanta, *n* = 1,217 synapses from 36 coverslips). Chi-squared test. **p < 0.01, ****p< 0.0001.

In the first experimental application of our new method, we wanted to confirm an ability to detect changes in mini frequency under conditions where this is known to change. We, therefore, applied nicotine (20 μM), well documented to cause robust increases in mini frequency (Gray et al., 1996; Sharma and Vijayaraghavan, 2003). Cultured neurons expressing SypH 2× were imaged as before in an experimental buffer containing nicotine. Background adjusted responses were plotted as frequency distribution, and four Gaussians were fitted as before (Figure 3A). The mean values of each Gaussian were plotted to reveal an evenly spaced distribution (*R^2^* = 0.97), with *q* = 0.44 ΔF/F_NH_4_Cl_, similar to that of control (Figure 3B).

To investigate changes in the frequency of minis between conditions, the fraction of boutons undergoing different numbers of release events (*q* = 0, *q* = 1, etc.) was assessed. The boundaries of each pair of Gaussians were calculated, and boutons that responded within each range were assigned as corresponding to that number of release events (Figure 3E). When nicotine was applied the data right-shifted as compared to control, with more boutons showing spontaneous release over the two minutes (Figure 3E). This confirms that our technique can report changes in mini frequency.

We then applied the technique to an investigation of changes in mini frequency following application of the Alzheimer’s-associated peptide Aβ in its most pathogenic (oligomeric) form (Aβo; Viola and Klein, 2015). This is an important question as miniature transmission may regulate synaptic strength (Sutton et al., 2006), which is reduced *via* poorly-understood mechanisms following Aβo treatment (Sheng et al., 2012). Previous studies examining the effects of Aβo on minis have performed electrophysiological recordings and conclude that mini frequency in cultured hippocampal neurons is reduced by Aβo treatment (Kamenetz et al., 2003; Shankar et al., 2007; Nimmrich et al., 2008; Parodi et al., 2010; Talantova et al., 2013). However, Aβo causes rapid postsynaptic depression and weakening (Li et al., 2009) that may reduce the number of electrically detectable events. We hypothesized that this might have confounded the electrophysiological data.

Cultured neurons expressing SypH 2× were incubated in media containing Aβo (200 nM) for over an hour and miniature release events were then measured as described above with the Aβo concentration maintained in the experimental buffer. Once again, responses were plotted as a frequency distribution in the same range as the control, and four Gaussians were objectively fitted (Figure 3C). The mean values of each Gaussian were plotted to find an evenly spaced distribution (*R^2^* = 0.95) with *q* = 0.037 ΔF/F_NH_4_Cl_ (Figure 3D), identical to that of control.

We then used the fraction of boutons undergoing different numbers of release events (*q* = 0, *q* = 1, etc.) to investigate changes in mini frequency as before (Figure 3F). As with our nicotine control, this revealed that the Aβo condition was right-shifted compared to control, with more boutons showing spontaneous release over 10 min, and more release events evident from those boutons. Chi-squared analysis of these results compared to control confirmed that the differences were highly significant (Figure 3F).

### Electrophysiological Recording of Miniature Release Shows a Decrease in Frequency Upon Aβo Incubation

**Figure 4:**
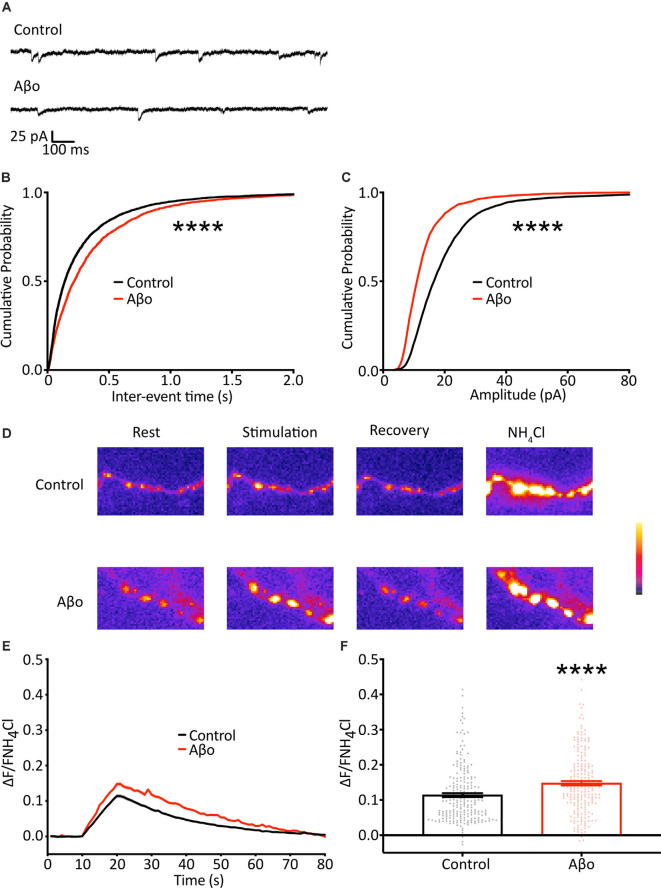
Electrophysiological recordings of mEPSCs show a reduction in frequency and amplitude following Aβo treatment. **(A)** Representative traces of mEPSC recordings. **(B)** Cumulative frequency distribution of mEPSC frequency. Kolmogorov-Smirnov test. **(C)** Cumulative frequency distribution of mEPSC amplitude. Kolmogorov-Smirnov test. **(D)** SypH 2×-expressing neurons were subjected to a 10 Hz/10 s stimulus train with or without prior incubation in Aβo. Panels show representative fluorescence images at stages of the experiment as indicated. **(E)** Average SypH 2× fluorescence traces. Shading represents ± SEM. **(F)** Mean peak amplitudes of responses ± SEM: control 0.114 ± 0.005, *n* = 218 synapses from seven coverslips; Aβo 0.148 ± 0.006, *n* = 212 synapses from six coverslips. Student’s *t*-test, ****p < 0.0001.

As the result of using the optical analysis approach is different from electrophysiological studies that report reduced mini frequency with Aβo, we wanted to ensure that this was not simply a reflection of differences in our experimental preparation. We therefore sought to replicate previous studies and recorded mEPSCs in hippocampal neurons electrophysiologically. Recordings were performed under voltage-clamp conditions, with the cell held at −70 mV, taking steps to maximize the sensitivity of mEPSC detection, including the use of a high impedance (low noise) amplifier and a cesium-containing internal solution to increase input resistance (Spruston et al., 1993; Fleidervish and Libman, 2008). In contrast to the increase in mini frequency we observed using direct optical measurements, we found a small reduction in mEPSC frequency (Figures 4A,B). Also, we found a decrease in mEPSC amplitude, consistent with Aβo-induced synaptic depression (Figure 4C). These data are similar to those reported by others performing electrophysiological recordings and suggest that our experimental preparation is comparable, and the effects of Aβo treatment are an appropriate context in which to test our optical method.

### Aβo Incubation Causes an Increase in Evoked Release

There is evidence both at the *Drosophila* neuromuscular junction (NMJ) and at mammalian central synapses that upon partial or complete blockage or loss of neurotransmitter receptors at the postsynaptic terminus (reduction in quantal size) there is a swift increase in the probability of evoked presynaptic release as a result of mechanisms of homeostatic synaptic plasticity (Pozo and Goda, 2010; Turrigiano, 2012; Davis and Muller, 2015). Here, we show a reduction in quantal size, evident as a decrease in mini amplitude, following Aβo incubation (Figure 4C), and we hypothesized that, in addition to the changes in the miniature release we describe above, the reduction in quantal size might elicit a homeostatic increase in evoked neurotransmitter release. Accordingly, we imaged cultured neurons expressing SypH 2× with and without incubation in Aβo (200 nM) and delivered 100 APs at 10 Hz to examine changes in evoked release (Figure 4D). We found that Aβo incubation potentiated release by about 30% compared to control (Figures 4E,F). This would be in keeping with a compensatory increase in the evoked release, although other interpretations, such as a direct effect of Aβo on the presynaptic terminal, remain possible.

## Discussion

We have developed a new optical technique for observing spontaneous miniature release events that allows direct detection of release, rather than relying on traditional, indirect measurements at the postsynaptic terminus such as the recording of mEPSCs. We found that this method gave a consistent ΔF/F_NH_4_Cl_ value for q across experimental conditions that was similar to that found with a one AP evoked stimulus, arguing in favor of its ability to detect quantal release events. To further validate the method, we investigated Pr in response to a one AP stimulus and found that it was comparable to that of previous studies (Branco et al., 2008).

In addition to pHluorin probes such as SypH 2×, several other methods of directly observing release at the presynaptic terminal have been developed. In particular, the fluorescent FM dyes have an inherently high signal and have been used to good effect to image presynaptic release in several systems (Kavalali and Jorgensen, 2014), including optical quantal analysis in cultured neurons (Tokuoka and Goda, 2008). New, optimized variants of the genetically encoded glutamate reporter iGluSnFR are also promising, as they allow rapid visualization of glutamate release both *in vitro* and *in vivo* (Hires et al., 2008; Marvin et al., 2013, 2018; Hefendehl et al., 2016; Xie et al., 2016; Helassa et al., 2018). While either of these techniques could, in principle, also be used to develop an optical quantal analysis of minis, they both have disadvantages: FM dyes are not genetically encoded, and therefore cannot be used to label genetically defined subpopulations as SypH 2× can. The iGluSnFR probe is genetically encoded, but as a glutamate sensor detection could be confounded by any change in the dynamics of glutamate concentration, including alterations to the rate of clearance by synaptic transporters. Nonetheless, it would be valuable to develop such tools for optical analysis of minis, not least as it offers a means of validation for results obtained using pHluorin probes.

Having established our optical measurement of minis, we demonstrated that the technique can be used to show alterations in spontaneous miniature release in cultured neurons, and critically reveal that changes not detected electrophysiologically in neurons exposed to the AD-associated peptide Aβ can be unmasked using our new approach. This outcome has particular significance as previous studies have shown that the addition of the pathogenic, oligomeric form of Aβ leads to postsynaptic weakening at an early stage in *in vitro* AD models, possibly *via* mechanisms of LTD leading to AMPAR internalization (Shankar et al., 2007, 2008; Li et al., 2009). Similar postsynaptic weakening seems to be evident at an early stage *in vivo* (Chang et al., 2006; Whitcomb et al., 2015; Zhang et al., 2018). Because minis are a key regulator of postsynaptic strength (Sutton et al., 2006), it is important to understand changes in mini frequency in the presence of Aβo. It is also important as an addition to our overall understanding of presynaptic regulation in AD. There is a growing body of evidence, which includes this study, that Aβo enhances evoked presynaptic release (Brito-Moreira et al., 2011; Russell et al., 2012; Lazarevic et al., 2017), although this is somewhat controversial (Nimmrich et al., 2008; He et al., 2019). At the same time, most studies that have examined mEPSC frequency in the presence of Aβo show that, in contrast to the changes in evoked release, it is reduced (Kamenetz et al., 2003; Shankar et al., 2007; Nimmrich et al., 2008; Talantova et al., 2013). This is puzzling since evoked and miniature release are typically tightly correlated; indeed, for this reason, mini frequency is often used as an indicator of overall presynaptic strength (Prange and Murphy, 1999). Our result helps resolve this apparent contradiction and suggests that Aβo does indeed enhance presynaptic function globally.

It is possible that enhanced neurotransmitter release (spontaneous and/or evoked) might result from the induction of homeostatic plasticity in response to postsynaptic weakening caused by Aβo, as can occur rapidly when the postsynaptic terminus is blocked or pharmacologically suppressed (Davis and Muller, 2015; Delvendahl et al., 2019). However, synaptic activity can itself homeostatically regulate postsynaptic strength on relatively short timescales (Ibata et al., 2008), and it remains possible that changes in presynaptic function are a primary event. Further work will be required to verify either these or alternative, possibilities.

It is increasingly apparent that regulation of presynaptic function is critical in mechanisms of learning and memory, and that presynaptic dysregulation can play an early role in a host of neurological disorders, including neurodegenerative diseases such as Parkinson’s disease and AD (Waites and Garner, 2011). Our findings add to the repertoire of techniques available to probe these important processes, as well as providing specific insight into the dysregulation of miniature neurotransmission in AD.

## Data Availability Statement

The raw data supporting the conclusions of this article will be made available by the authors, without undue reservation.

## Ethics Statement

Ethical review and approval was not required for the animal study because *in vivo* work was not performed in this study, and all procedures were carried out in accordance with the Animals (Scientific Procedures) Act, 1986 (UK).

## Author Contributions

HT and AJ planned and designed the study. HT performed the experiments. RT assisted with coding and analysis of SypH 2× data. AJ and NE provided oversight for the work. All authors contributed to the article and approved the submitted version.

## Conflict of Interest

The authors declare that the research was conducted in the absence of any commercial or financial relationships that could be construed as a potential conflict of interest.
